# Risk factors for further recurrences of *Clostridioides difficile* infection at the first and second recurrence: a retrospective cohort

**DOI:** 10.1186/s12879-025-11495-0

**Published:** 2025-08-22

**Authors:** Måns Stefansson, Oscar Bladh, Piotr Nowak, Lars Rombo, Magnus Hedenstierna, Johan Ursing

**Affiliations:** 1 Department of Clinical Sciences Danderyd Hospital, Karolinska Institutet, Stockholm, Sweden; 2 Centre for Clinical Research Sörmland, Uppsala University, Eskilstuna, Sweden; 3 Department of Infectious Diseases, Karolinska University Hospital, Huddinge, Stockholm, Sweden; 4 Centre for Infectious Medicine, Department of Medicine Huddinge, Karolinska Institutet, Stockholm, Sweden; 5 Department of Infectious Diseases, Danderyd Hospital, Stockholm, Sweden

**Keywords:** *Clostridioides difficile*, Recurrent *Clostridioides difficile* infection

## Abstract

**Background:**

Recurrent *Clostridioides difficile* infection is most effectively treated with faecal microbiota transplantation. Swedish and European guidelines suggest faecal microbiota transplantation after a first or second recurrence, respectively. The aims of this study were to evaluate risk factors for further relapses at the first and second recurrence, related to treatment recommendations.

**Methods:**

Patients aged ≥ 18 years with two positive tests for *C. difficile* within eight weeks and treated at the study hospitals during 2014–2022 were eligible for inclusion. Retrospectively collected data included age, sex, treatment, and clinical characteristics for each episode. Risk factors for further recurrences at the first and second recurrence were identified using multivariable logistic regression analysis.

**Results:**

The median age in the total cohort (*n* = 231) was 76 (IQR 67–84) years, 52% were females and 15% were healthy without comorbidities. One recurrence only occurred in 110 patients (48%), however, no clinically significant risk factors predicting more than one recurrence were identified. Two or more recurrences occurred in 110 patients, of whom, 44 (40%) had further recurrences. Frailty (Clinical Frailty Scale ≥ 4) was significantly associated with more than two recurrences (*p* = 0.03). The respective median times between the first and the second recurrences were 12 and 17 days in patients with more than two recurrences compared to two recurrences only (*p* = 0.02).

**Conclusions:**

Patients experiencing a second recurrence of *C. difficile* infection who were frail and relapsed in a shorter time span after the first recurrence had a significantly increased risk of further recurrences.

**Supplementary Information:**

The online version contains supplementary material available at 10.1186/s12879-025-11495-0.

## Background

*Clostridioides difficile* infection (CDI) is a major healthcare-associated infection with an estimated 462 100 cases in the US and 190 000 hospital-acquired infections in the EU/European economic area in 2017 [1, 2]. The proportion of hospital-acquired CDI differ between countries, 92% of CDI cases in Sweden between 2006 and 2019 were estimated to be hospital-acquired compared to a recent estimation of 51% healthcare-associated infections in the US in 2017 [1, 3]. Moreover, Swedish patients with a history of CDI had a nine times higher all-cause hospital-admission rate compared to matched controls [3].

Risk factors for a first episode of CDI are healthcare exposure, comorbidities (predominately kidney failure), and antibiotic treatment [4, 5]. Clindamycin and cephalosporins are the antimicrobials associated with the highest risk of CDI [6]. Recurrent CDI (rCDI) is defined as diarrhoea or colitis and a positive test within eight weeks of a previous episode [7]. The risk of rCDI after a first episode is estimated to be 5–60%, with a median of 20% [8, 9]. The risk of further recurrences after a first recurrence (second episode) is 25–65% [10, 11]. Elderly persons with multiple comorbidities and healthcare exposure have the greatest risk of recurrence [5, 12].

The antibiotics vancomycin and fidaxomicin are effective for both primary and rCDI. Metronidazole has a lower efficacy and higher recurrence rates and is therefore no longer a first-hand choice [13]. The most commonly used preventive treatment for rCDI is faecal microbiota transplantation (FMT), monoclonal antibodies directed at toxin B were recommended but are no longer available [13–15]. FMT is the most effective treatment for recurrent disease with cure rates up to 90–95% and was previously recommended after multiple recurrences, but only given to approximately 10% of eligible patients [16–18]. The current European guidelines recommend FMT after two recurrences whilst Swedish national guidelines suggests FMT after the first recurrence [13, 19]. It is not possible to treat all patients with rCDI according to the recommendations due to limited FMT availability. Risk stratification of patients with rCDI to prioritize FMT to those at increased risk of recurrence is therefore necessary. The primary aim of this study was to identify risk factors for a second recurrence in a cohort of patients with a first recurrence. A secondary aim for the population of patients with two recurrences or more, was to identify risk factors associated with more than two recurrences (≥ 3 recurrences) compared to only two recurrences of CDI.

## Methods

### Study design and setting

This was a multicentre, retrospective, cohort study of patients with rCDI. Study sites were the secondary care hospitals Norrtälje and Danderyd hospitals in north-eastern Stockholm County, with approximately 600 beds in total and servicing a combined population of around 650 000 people. The study was performed following the STROBE guidelines.

### Identification of patients

Patients for inclusion were identified through microbiological records of positive test results for *C. difficile* provided by the Department of Microbiology, Karolinska University Hospital and from records of patients treated for rCDI at the Department of Infectious Diseases, Danderyd Hospital. The testing methods at the laboratory were PCR for detection of toxin B DNA (TcdB) (GenomEra CDX System, ABACUS Diagnostica) or multiplex RT PCR of the toxin B gene (TcdB) (GeneXpert Dx System, Cepheid). Electronic medical records of the identified patients were examined retrospectively. The study period was from 1 st of September 2014 until 31 st December 2022.

Inclusion criteria were recurrent CDI, defined as diarrhoea and a positive test for CDI, followed by resolution of symptoms and subsequent recurrence of diarrhoea and a second positive test within 66 days (eight weeks plus 10 days of standard treatment). Following episodes with the same characteristics were included if the time from completed treatment until a new positive test and symptoms was eight weeks or less.

Exclusion criteria were age below 18 years, chronic vancomycin treatment after the first recurrence, or death during follow up before a subsequent recurrence could occur. Individuals who had undergone FMT treatment for the first recurrence were also excluded from the cohort, as they were likely to have had previous episodes treated by another caregiver.

### Definitions and variables

CDI was defined as an episode of diarrhoea with a concurrent positive *C. difficile* stool sample that was treated as CDI, according to the medical notes. CDI severity was graded as non-severe or severe. Severe CDI was defined as the occurrence of one of the following parameters based on the ESCMID guidelines published in 2021 [13]: white blood cell count > 15 × 10^9^/L, creatinine > 130 µmol/L, fever > 38.5^°^C or shock. A recurrence was defined as a new episode with recurrent symptoms of CDI within eight weeks after completing treatment for an earlier episode [13].

The medical records were examined manually, and data was recorded in REDCap™. Baseline data at the first episode were age, sex, body mass index, comorbidities, frailty, concomitant treatment with prespecified drugs, antibiotic exposure and hospital admissions, the latter two parameters during the past 90 days. Comorbidities were defined by ICD-10 codes or a written description, and evaluated with Charlson Comorbidity Index [20]. Frailty was described according to Clinical Frailty Scale [21]. The investigated medications included immunosuppressant drugs, opioids, and proton pump inhibitors (PPIs). Immunosuppressive treatment was exemplified as treatment with at least 10 mg prednisolone daily for more than four weeks, current chemotherapy (including methotrexate), anti-CD20 antibodies (rituximab) or inhibitors of tumour necrosis factor-alpha.

For every episode, the following factors were determined: treatment for CDI, hospitalization for the present episode, CDI severity, levels of C-reactive protein (CRP, mg/L), white blood cell (WBC) count (x10^9^/L), and creatinine (µmol/L).

Metronidazole was the recommended treatment for a first episode of CDI until the year 2021, when the recommendation was changed to fidaxomicin or vancomycin [13]. Faecal microbiota transplantation was performed after multiple recurrences according to national guidelines in use until 2021. The FMT treatment was either non-pooled, donated faeces from related and unrelated donors, or cultured microbiota provided from a microbiological laboratory [22].

### Statistics

Categorical variables were reported as proportions and continuous variables as medians with interquartile ranges (IQR). The cohort was divided into groups with only one recurrence (two episodes) compared to the group with more than one recurrence (three episodes or more). We also assessed the subcohort of patients experiencing two recurrences only (three episodes) or more than two recurrences (four or more episodes). Persons that died or were prescribed chronic vancomycin treatment after the second recurrence were excluded from the subcohort. Clinical Frailty Scale was divided into groups of persons that were at least managing well (CFS 1–3) or that were very mildly frail to terminally ill (CFS ≥ 4) and was analysed as a categorical variable.

Differences for categorical variables between the groups were assessed using Chi-square test, or for small numbers, Fisher’s exact test. Continuous variables were compared using nonparametric testing of independent samples with Mann-Whitney U-test. Univariable followed by multivariable logistic regression analyses were performed. Predictors were included in the multivariable analysis if the *p*-value in the univariable analysis was < 0.1. The logistic regression analysis was performed for the total cohort regarding predictors associated with one versus more than one recurrence of CDI, and for the subcohort that compared predictors associated with two versus more than two recurrences of CDI. Receiver operating characteristics (ROC) curve, negative and positive predictive values were calculated for relevant predictors including 95% confidence intervals. A p-value < 0.05 was considered significant. The statistical software SPSS version 29.0.2.0 was used for the statistical calculations (20).

## Results

**Fig. 1:**
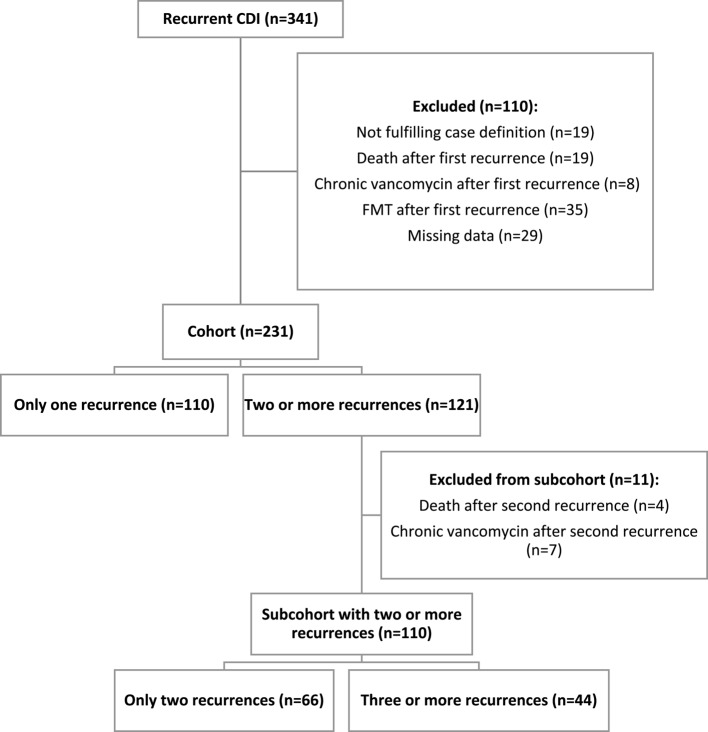
Flowchart of patients included in the cohort and subcohort

A total of 1440 patients had at least one positive test for *Clostridioides difficile* at the study hospitals during the study period. Of these, 24% (341/1440) had at least one suspected recurrence of CDI, and 231 were included in the final analysis (Fig. 1). One hundred and ten patients were excluded due to missing data and other factors as seen in the flowchart. 48% (110/231) had one recurrence only and the remaining 52% (121/231) had more than one recurrence. Eleven of the 121 patients died or received chronic treatment with vancomycin after the second recurrence. These patients were excluded from the subcohort of patients with two or more recurrences (*n* = 110).

### Baseline characteristics and comparison of patients with one or more than one recurrent episode of CDI

The median age in the cohort was 76 (IQR 67–84) years and 52% of the patients were females (Table 1). 85% had received antibiotic treatment, and 63% had been hospitalized within ninety days prior to the first episode. Only 15% were deemed healthy without any comorbidities. Baseline characteristics such as age, sex, Clinical Frailty Scale, median Charlson Comorbidity Index, and severity of the first episode did not differ significantly between the groups with one (*n* = 110) versus more than one recurrence (*n* = 121) (Table 1). The type of antibiotic treatment before the primary episode and use of PPIs and other investigated drugs were not significantly associated with risk of multiple recurrences and are reported in Additional File 1.

**Table 1:** Baseline characteristics

	All	One recurrence	More than one recurrence	*p*-value
	*n* (%) or median (IQR)			
Total	231	110 (48)	121 (52)	
*Sex*				0.49
Female	121 (52)	55 (50)	66 (55)	
Male	110 (48)	55 (50)	55 (45)	
Age (years)	76 (67–84)	78 (64–86)	76 (68–82)	0.5
Body Mass Index	24 (21–27)	24 (21–27)	24 (20–27)	0.47
Clinical Frailty Scale (CFS) 4–9	143 (70)	67 (74)	76 (68)	0.37
Charlson comorbidity index	2 (0–4)	2 (1–3)	2 (0–4)	0.54
Healthy	35 (15)	14 (13)	21 (17)	0.33
Hypertension	111 (48)	47 (43)	64 (53)	0.12
Cardiac disease	63 (27)	28 (26)	35 (29)	0.55
Kidney failure GFR < 30 or dialysis	19 (8)	9 (8)	10 (8)	0.98
Liver failure	15 (7)	7 (6)	8 (7)	0.94
Cancer	40 (17)	16 (15)	24 (20)	0.29
Cerebrovascular disease	40 (17)	27 (25)	13 (11)	**0.01**
Diabetes	45 (20)	26 (24)	19 (16)	0.13
Cholecystectomy	7 (3)	3 (3)	4 (3)	0.8
Immunosuppression	42 (18)	15 (14)	27 (22)	0.09
Antibiotics last 90 days	196 (85)	93 (85)	103 (85)	0.9
Antibiotic treatment duration (days)	11 (8–20)	12 (8–24)	11 (8–17)	0.48
Hospitalized last 90 days	145 (63)	70 (64)	75 (63)	0.85
Characteristics of 1^st^ episode of CDI				
Hospitalized	153 (66)	81 (75)	72 (60)	**0.02**
Severe CDI	82 (35)	40 (38)	42 (38)	0.95
Days between 1^st^ CDI and 1^st^ recurrence	11 (8–18)	11 (7–18)	12 (8–18)	0.72
Treatments at 1^st^ recurrence of CDI				
Metronidazole	68 (29)	36 (33)	32 (26)	0.3
Vancomycin	130 (56)	58 (53)	72 (60)	0.3
Combination	33 (14)	16 (15)	17 (14)	0.91
Treatment duration (days)	11 (10–17)	11 (10–20)	10 (10–15)	0.48
Characteristics of 1^st^ recurrence of CDI				
C-reactive protein (mg/L)	55 (22–111)	62 (26–114)	54 (19–109)	0.43
White Blood Cell count (x10 ^9^ /L)	12 (8–18)	12 (7–18)	13 (9–18)	0.44
Creatinine (µmol/L)	80 (61–114)	73 (58–116)	81 (64–108)	0.57
Hospitalized	109 (52)	48 (50)	61 (53)	0.61
Severe CDI	45 (25)	18 (23)	27 (26)	0.59

Missing data at baseline or first episode: Body Mass Index (*n* = 9), CFS (*n* = 28), antibiotics last 90 days (*n* = 1), hospitalized last 90 days (*n* = 2), antibiotic days (*n* = 48), C-reactive protein (*n* = 44), White Blood Cell count (*n* = 47), creatinine (*n* = 48), hospitalized for index CDI (*n* = 3), severity (*n* = 15)

Missing data for 1 st recurrence: C-reactive protein (*n* = 115), White Blood Cell count (*n* = 116), creatinine (*n* = 119), hospitalized for 1 st recurrence (*n* = 21), severity (*n* = 47)

Percentages for variables with missing values are valid percentage, excluding the missing values

*IQR* Interquartile Range, *CDIClostridioides difficile* infection, *GFR* Glomerular Filtration Rate (mL/min/1.73 m²)

In the multivariable analysis, patients with cerebrovascular disease (OR 0.39, 95% CI 0.19–0.81) and hospitalization for the first episode (OR 0.54, 95% CI 0.3–0.96) had a significantly lower risk of more than one recurrence (Table 2). Nonparametric testing for age distribution amongst persons diagnosed with stroke compared to no stroke showed no difference. Described risk factors for multiple recurrences, like previous hospitalization and older age, are displayed in the univariable analysis but the significance did not meet the prespecified value of p = < 0.1 for inclusion in the multivariable analysis.

**Table 2:** Logistic regression analysis

	Total cohort				Subcohort with two recurrences of CDI			
	Univariable analysis		Multivariable analysis		Univariable analysis		Multivariable analysis	
	Odds ratio (95% CI)	*p*-value	Odds ratio (95% CI)	*p*-value	Odds ratio (95% CI)	*p*-value	Odds ratio (95% CI)	*p*-value
Age	1 (0.99–1.02)	0.91			1.02 (0.99–1.05)	0.29		
Female	1.2 (0.72–2.01)	0.49			1.06 (0.49–2.31)	0.88		
Hospitalized last 90 days	0.95 (0.55–1.63)	0.85			1.21 (0.54–2.73)	0.64		
Clinical Frailty Scale 4–9	0.76 (0.41–1.4)	0.37			2.92 (1.16–7.32)	0.02	2.92 (1.11–7.67)	**0.03**
Charlson Comorbidity Index	0.98 (0.88–1.1)	0.76			1.16 (0.98–1.39)	0.09		
Healthy	1.44 (0.69–2.99)	0.33			0.31 (0.1–1.01)	0.05		
Kidney failure GFR < 30	1.01 (0.4–2.59)	0.98			3.31 (0.78–14.04)	0.1	3.89 (0.8-18.85)	0.09
Cerebrovascular disease	0.37 (0.18–0.76)	0.01	0.39 (0.19–0.81)	**0.01**	1.08 (0.32–3.65)	0.9		
Immunosuppression	1.82 (0.91–3.64)	0.09	1.61 (0.79–3.29)	0.19	0.92 (0.37–2.27)	0.86		
Carbapenems	0.9 (0.33–2.49)	0.84			5.05 (0.97–26.31)	0.05	5.09 (0.78–33.27)	0.09
Hospitalized for 1^st^ CDI	0.5 (0.28–0.88)	0.02	0.54 (0.3–0.96)	**0.02**	0.73 (0.38–1.59)	0.43		
Days between 1^st^ and 2^nd^ recurrence of CDI					0.96 (0.93-1)	0.03	0.95 (0.92–0.99)	**0.02**
C-reactive protein (mg/L) at 2^nd^ recurrence					0.99 (0.98-1)	0.02		

Regression analyses for total cohort (one versus more than one recurrence) and the subcohort (two versus more than two recurrences). The predictors Healthy and Charlson Comorbidity Index were excluded from the multivariable analysis due to collinearity with Clinical Frailty Scale regarding a person’s general health and symptoms of chronic conditions. Age, sex and previous hospitalization are reported in the univariable analysis since they are previously described risk factors for recurrence. C-reactive protein (mg/L) at 2nd recurrence was excluded from the multivariable analysis due to a high extent of missing values (47%)

*CI* Confidence Interval, *GFR* Glomerular Filtration Rate (mL/min/1.73 m^2^), *CDIClostridioides* difficile infection

### Comparison of patients with two recurrences of CDI only versus more than two recurrences of CDI

One hundred ten patients had at least two recurrences and were analysed in the subcohort. 60% (66/110) had two recurrences only and 40% (44/110) had more than two recurrences (Table 3). Being frail (Clinical Frailty Scale of 4 or more) was significantly associated with further recurrences (OR 2.92, 95% CI 1.11–7.67) and longer time between the first and second recurrence was significantly associated with cure (OR 0.95, 95% CI 0.92–0.99) in the multivariable analysis (Table 2). C-reactive protein was significantly lower for patients with more than two recurrences in the univariable analysis but was not included in the multivariable analysis due to approximately 50% missing data. Health status and Charlson Comorbidity Index were excluded from the multivariable analysis since they are correlated to the predictor Clinical Frailty Scale, which include information and symptoms of chronic disease.

**Table 3:** Characteristics for the subcohort

	All	Two recurrences	More than two recurrences	*p*-value
	*n* (%) or median (IQR)			
Total	110	66 (60)	44 (40)	
*Sex*				0.88
Female	64 (58)	38 (58)	26 (59)	
Male	46 (42)	28 (42)	18 (41)	
Age (years)	75 (68–80)	75 (67–80)	76 (70–82)	0.47
Body Mass Index	23 (20–27)	24 (21–27)	22 (20–26)	0.17
Clinical Frailty Scale 4–9	69 (66)	37 (58)	32 (80)	**0.02**
Charlson comorbidity index	1 (0–4)	1 (0–3)	2 (0–4)	**0.03**
Healthy	20 (18)	16 (24)	4 (9)	**0.04**
Hypertension	57 (52)	35 (53)	22 (50)	0.75
Cardiac disease	27 (25)	14 (21)	13 (30)	0.32
Kidney failure GFR < 30 or dialysis	9 (8)	3 (5)	6 (14)	0.09
Liver failure	8 (7)	4 (6)	4 (9)	0.55
Cancer	20 (18)	12 (18)	8 (18)	1
Cerebrovascular disease	20 (18)	11 (17)	9 (21)	0.61
Diabetes	16 (15)	8 (12)	8 (18)	0.38
Cholecystectomy	4 (4)	1 (2)	3 (7)	0.15
Immunosuppression	26 (24)	16 (24)	10 (23)	0.86
Antibiotics last 90 days	95 (86)	57 (86)	38 (86)	1
Hospitalized last 90 days	70 (65)	41 (63)	29 (67)	0.64
Treatments at 2^nd^ recurrence				
Metronidazole	15 (14)	9 (14)	6 (14)	1
Vancomycin	40 (36)	22 (33)	18 (41)	0.42
FMT	44 (40)	30 (45)	14 (32)	0.15
Combination antibiotics	11 (10)	5 (8)	6 (14)	0.34
Treatment duration (days)	14 (10–30)	12 (10–27)	16 (10–35)	0.1
Characteristics of 2nd recurrence				
C-reactive protein (mg/L)	57 (25–120)	88 (30–168)	48 (17–83)	**0.03**
White Blood Cell count (x10^9^ /L)	12 (9–18)	11 (9–17)	13 (9–22)	0.37
Creatinine (µmol/L)	77 (55–124)	76 (50–99)	78 (58–146)	0.29
Hospitalized	48 (47)	27 (42)	21 (54)	0.25
Severe CDI	24 (25)	13 (22)	11 (30)	0.37
Days between 1^st^ and 2^nd^ recurrence of CDI	15 (9–26)	17 (10–29)	12 (6–19)	**0.003**

Missing data at baseline: Body Mass Index (*n* = 3), CFS (*n* = 6), hospitalized last 90 days (*n* = 2), antibiotic days (*n* = 23). Missing data at second recurrence: C-reactive protein (52), White Blood Cell count (54), creatinine (53), hospitalized for second recurrence (7), treatment days (6), severity (13)

Percentages for variables with missing values are valid percentage, excluding the missing values

*IQR* Interquartile Range, *CDIClostridioides difficile* infection, *GFR* Glomerular Filtration Rate (mL/min/1.73 m²)

#### ROC-curve, positive and negative predictive values for risk factors comparing two recurrences only, versus more than two recurrences

**Fig. 2:**
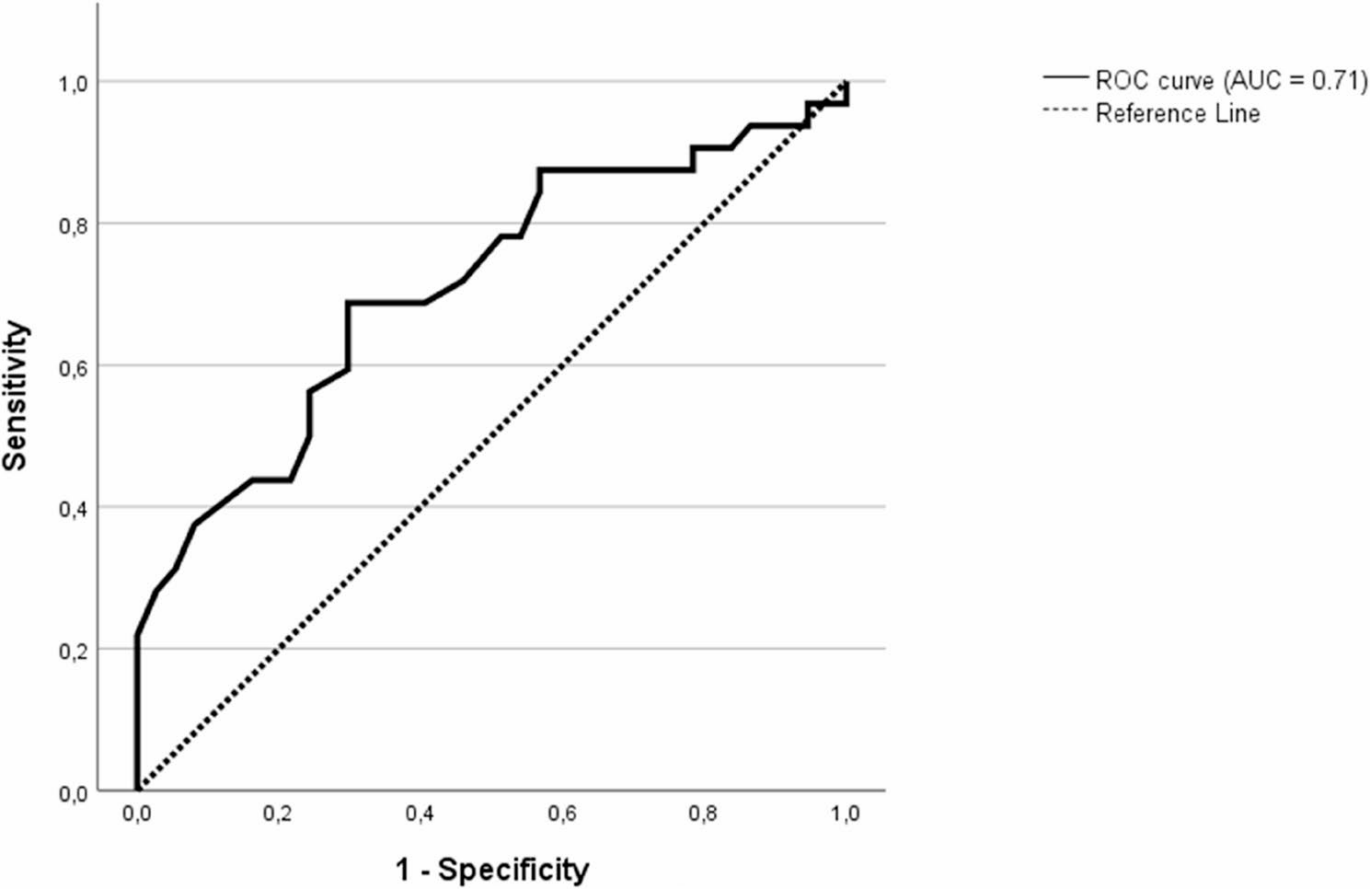
ROC curve for frail patients (CFS ≥ 4) describing time to the second recurrence as a predictor for more than two recurrences, shorter time until the second recurrence indicates more positive result. Area under the curve was 0.71 (95% CI 0.59–0.84, *p* = 0.001)

The negative predictive value for a third recurrence was 77% (95% CI 62–89) for persons that were considered not frail (Additional File 1, Supplementary Table 2). The positive predictive value for further recurrences was 63% (95% CI 46–79) for patients with CFS ≥ 4 that experienced the second recurrence less than two weeks after finishing treatment for the first recurrence (*n* = 19). The area under the ROC-curve to distinguish frail patients with only two compared to two or more recurrences regarding time to the second recurrence was 0.71 (95% CI 0.59–0.84, *p* = 0.001), as seen in Fig. 2.

## Discussion

Two hundred and thirty-one patients with rCDI were included in this cohort over the study period from 2014 to 2022. Of these, 52% (*n* = 121) experienced more than one recurrence, consistent with previously published data with multiple recurrence rates of 40–60% [10, 11, 23].

At the first recurrence, patients previously diagnosed with cerebrovascular disease had a significantly lower risk of a second recurrence (*p* = 0.01). Potential confounder such as age did not vary significantly for patients with or without cerebrovascular disease suggesting that the finding is correct for this cohort. Higher mortality was also not an explanation for this result since patients that died during follow up were excluded. Results in previous studies are not consistent, one study showed a lower proportion of patients with cerebrovascular disease in the group with only one compared to two recurrences and another study found no difference [23, 24]. Admission to hospital for the first CDI episode was also associated to one recurrence only (*p* = 0.02). The disease severity and treatment for the first episode were similar amongst hospitalized and non-hospitalized patients and did not explain this result or the reason for admission to hospital. Hospitalization for any other cause in the past 90 days did not increase the risk for more than one recurrence, unlike earlier reports, where this was significantly associated to multiple recurrences [10]. Known risk factors for rCDI, like sex, older age, exposure of high risk-antibiotics, and PPIs were evaluated without increased risk of a second recurrence. This corresponds to earlier research where risk factors, including haemodialysis and age > 65 years, were not significantly associated with an increased risk of multiple recurrences, except for in one study [10, 23, 24]. Thus, no useful risk factors for multiple recurrences were identified at the first recurrence to predict who would have a greater need for FMT.

The secondary aim of this study was to identify patients at risk for more than two recurrences of CDI, in line with international FMT treatment recommendations [13, 14]. 40% (*n* = 44) of the 110 patients in the subcohort with more than one recurrence experienced further recurrences. Being frail (Clinical Frailty Scale of 4 or more) and short time between the first and the second recurrence (median 12 compared to 17 days) were significantly associated with multiple recurrences. A previous study found that patients with more than two recurrences had a higher score of Charlson Comorbidity Index than patients with only two recurrences (mean score 2.3 versus 1.8, no significance testing reported), which is similar to our findings regarding Charlson Comorbidity Index and might correspond to higher frailty level [25]. Two other recent studies did not show any significant differences comparing these groups, but frailty was not evaluated [23, 24]. A possible explanation of our results is that frail persons have a less diverse and resilient gut microbiome, which leads to an increased risk of rCDI [26]. Persons with a higher frailty level also have more frequent healthcare contacts and are hence more exposed for both *C. difficile* and antibiotics [27, 28]. Consistent with our data, the majority of recurrences have previously been shown to occur within two weeks, with a reported median time from the second to the third recurrence of 9 days [23, 29]. Moreover, rapid recurrence is a strong indicator of relapse with the same strain, rather than a new infection caused by a new strain [29, 30]. Our results thus appear to correlate to previous data even though frailty and a rapid recurrence as risk factors for multiple recurrences rarely have been studied [23, 25].

The positive predictive value for a third recurrence was 63% for frail patients that relapsed with a second recurrence in two weeks or less after the first recurrence. Since multiple recurrences are known risk factors for both further recurrences and complications to rCDI, such as sepsis and colectomy, this suggests that these frail patients with a rapid relapse should be prioritized for FMT at the second recurrence [24, 25]. Conversely, patients with Clinical Frailty Score of 1–3 had a negative predictive value of 77% for further recurrences at the second recurrence, indicating that persons independent in their daily life had a lower risk of more than two recurrences and hence less to gain from FMT. These results underline the importance of determining risk factors for multiple recurrences, so that patients with the highest risk for further recurrences can receive the most effective treatment.

There is a number of limitations of this study, a major limitation is the lack of data on concomitant non-CDI antibiotics during the *C. difficile* infection and new antibiotic treatment prior to the registered recurrences. The regional testing algorithm favours testing with a PCR-based method without a confirming EIA-test, which might lead to an overdiagnosis of CDI due to carriage of *C. difficile* concomitant with other diarrheal diseases, but all episodes included were treated as CDI. There might have been previous, not identified, episodes of CDI treated by another caregiver since the microbiological records cover all but one of the hospitals in the region and not some private primary care centres. Retrospectively collected data is always associated with a certain degree of missing information but the distribution of missing data should be similar between the compared groups. Notable strengths of the study include the elderly study population with high antibiotic exposure and multiple comorbidities, similar to other studies suggesting that the results are generalisable [9, 31, 32].

## Conclusion

No clinically significant risk factors associated with further recurrences were found in a cohort of patients with one recurrence of CDI. Frailty and shorter time to the second recurrence were significantly associated with more than two recurrences compared to only two recurrences. Frail persons with a rapid relapse should be considered for FMT at the second recurrence.

## Supplementary Information

Supplementary Material 1.

## Data Availability

Data is currently not publicly available due to an existing key, which makes the dataset non-anonymized according to European General Data Protection Regulation. Reasonable request to access the data can be sent to the corresponding authors.

## Abbreviations

US: United States
EU: European Union
CDI: Clostridioides difficile infection
rCDI: Recurrent Clostridioides difficile infection
FMT: Faecal microbiota transplantation
PCR: Polymerase Chain Reaction
RT PCR: Reverse Transcription Polymerase Chain Reaction
ESCMID: European Society of Clinical Microbiology and Infectious Diseases
PPIs: Proton Pump Inhibitors
CRP: C-reactive protein
WBC: White blood cell
IQR: Interquartile ranges
CFS: Clinical Frailty Scale
GFR: Glomerular Filtration Rate
CI: Confidence Interval
PPV: Positive predictive value
NPV: Negative Predictive Value
