# Supplementary material for: Risk factors for further recurrences of Clostridioides difficile infection at the first and second recurrence: a retrospective cohort
Source: BMC Infect Dis. 2025 Aug 22;25:1057. doi: 10.1186/s12879-025-11495-0 (PMC12372216; doi:10.1186/s12879-025-11495-0)
Supplement: Supplementary file 1 — Supplementary Material 1. [file 12879_2025_11495_MOESM1_ESM.docx]

| Additional File 1 **Supplementary table 1** | | |  | |  | | |  | | |  |
| --- | --- | --- | --- | --- | --- | --- | --- | --- | --- | --- | --- |
| **Total cohort** | | | | | | | **Subcohort with two or more recurrences of CDI** | | | | |
|  | **All** | **One recurrence** | | **More than one recurrence** | | **p-value** | **Subcohort all** | | **Two recurrences** | **More than two recurrences** | **p-value** |
|  | **n (%) or median (IQR)** | | | | |  | **n (%) or median (IQR)** | | | | |
| **Penicillins** | 101 (44) | 43 (39) | | 58 (48) | | 0.18 | 53 (48) | | 33 (50) | 20 (46) | 0.64 |
| **Ciprofloxacin** | 48 (21) | 24 (22) | | 24 (20) | | 0.71 | 21 (19) | | 13 (20) | 8 (18) | 0.84 |
| **Cephalosporins** | 76 (33) | 41 (37) | | 35 (29) | | 0.18 | 30 (27) | | 20 (30) | 10 (23) | 0.38 |
| **Carbapenems** | 16 (7) | 8 (7) | | 8 (7) | | 0.84 | 8 (7) | | 2 (3) | 6 (14) | **0.04** |
| **TMP-SMX** | 21 (9) | 9 (8) | | 12 (10) | | 0.65 | 10 (9) | | 3 (5) | 7 (16) | 0.09 |
| **Nitrofurantoin** | 3 (1) | 2 (2) | | 1 (1) | | 0.6 | 1 (1) | | 0 | 1 (2) | 0.4 |
| **Clindamycin** | 68 (29) | 29 (26) | | 39 (32) | | 0.33 | 37 (34) | | 23 (35) | 14 (32) | 0.74 |
| **Piperacillin/TZB** | 50 (22) | 24 (22) | | 26 (22) | | 0.95 | 25 (23) | | 14 (21) | 11 (25) | 0.64 |
| **PPIs** | 97 (42) | 45 (41) | | 52 (43) | | 0.75 | 46 (42) | | 28 (42) | 18 (41) | 0.88 |
| **Opioids** | 46 (20) | 22 (20) | | 24 (20) | | 0.98 | 21(19) | | 13 (20) | 8 (18) | 0.84 |
| **Metformin** | 6 (3) | 3 (3) | | 3 (3) | | 1 | 3 (3) | | 2 (3) | 1 (2) | 1 |
| ***First CDI*** |  |  | |  | |  |  | |  |  |  |
| **Metronidazole** | 178 (77) | 87 (79) | | 91 (75) | | 0.48 |  | |  |  |  |
| **Vancomycin** | 19 (8) | 8 (7) | | 11 (10) | | 0.62 |  | |  |  |  |
| **Combination** | 32 (14) | 14 (13) | | 18 (15) | | 0.64 |  | |  |  |  |
| **No treatment** | 2 (1) | 1 (1) | | 1 (1) | | 1 |  | |  |  |  |
| **Treatment duration (days)** | 10 (10-11) | 10 (10-11) | | 10 (10-11) | | 0.76 |  | |  |  |  |
| **C-reactive protein (mg/L)** | 76 (28-137) | 82 (34-147) | | 63 (25-130) | | 0.23 |  | |  |  |  |
| **White Blood Cell count (x10^9^/L)** | 12 (8-18) | 12 (8-18) | | 12 (9-19) | | 0.58 |  | |  |  |  |
| **Creatinine (µmol/L)** | 84 (59-128) | 85 (59-125) | | 82 (60-138) | | 0.7 |  | |  |  |  |

**IQR: Interquartile Range; CDI: *Clostridioides difficile* infection; TMP-SMX: Trimethoprim/sulfamethoxazole; TZB: Tazobactam; PPIs: Proton Pump Inhibitors**

**Legend Supplementary Table 1: Pre-index treatment with antimicrobials and other drugs, and clinical characteristics of the index episode for the cohort and subcohort.** Penicillins include e.g. benzylpenicillin, amoxicillin and cloxacillin

**Supplementary Table 2: Positive and negative predictive value for a third recurrence**

|  |  | |  |  |
| --- | --- | --- | --- | --- |
|  | **Two recurrences (n)** | **More than two recurrences (n)** | **PPV (95% CI)** | **NPV (95% CI)** |
| **Frail (CFS ≥4)** | 37 | 32 | 46% (35-58) | 77% (62-89) |
| **Not frail (CFS 1-3)** | 27 | 8 |  |  |
| **<15 days between 1^st^ - 2^nd^ recurrence** | 25 | 27 | 52% (39-65) | 71% (58-81) |
| **>15 days between 1^st^ - 2^nd^ recurrence** | 41 | 17 |  |  |
| **Frail and <15 days 1^st^ - 2^nd^ recurrence** | 11 | 19 | 63% (46-79) | 69% (58-78) |
| **Non-frail and >15 days 1^st^ - 2^nd^ recurrence** | 55 | 25 |  |  |
| **PPV: Positive predictive value; NPV: Negative Predictive Value; CI: Confidence Interval; CFS: Clinical Frailty Scale**  **Legend Supplementary Table 2: Positive and negative predictive value for a third recurrence depending on frailty and time between first and second recurrence.** Frail persons were defined as a Clinical Frailty Scale of 4 or more. Rapid recurrence was defined as a recurrence within two weeks after completion of treatment for the previous episode. | | | | |
